# Multiple event monitoring

**DOI:** 10.1186/s41235-016-0022-7

**Published:** 2016-12-12

**Authors:** Chia-Chien Wu, Jeremy M. Wolfe

**Affiliations:** 1 Harvard Medical School, Boston, USA; 2 Visual Attention Lab, Brigham and Women’s Hospital, Boston, USA

**Keywords:** Tracking Performance, Visual Working Memory, Position Tracking, Tracking Accuracy, Event Monitoring

## Abstract

Suppose you were monitoring a group of people in order to determine if anyone of them did something suspicious (e.g., putting down a bag) or if any two interacted in a suspicious manner (e.g., trading bags). How large a group could you monitor successfully? This paper reports on six experiments in which observers monitor a group of entities, watching for an event. Whether the event was performed by a single entity or was an interaction between a pair, the capacity for event monitoring was two to three items. This was lower than the multiple object tracking capacity for the same stimuli (approximately six items). Capacity was essentially the same whether entities were identical circles or unique cartoon animals; nor was capacity changed by an added requirement to identify the entities involved in an event. Event monitoring appears to be related to, but not identical to, multiple object tracking.

## Significance

In a surveillance situation, an individual might be required to monitor a crowd and look for a suspicious event among them (e.g., Did anyone abandon a bag?). Similarly, a lifeguard might monitor a pool for swimmers in danger. What is our capacity for “event monitoring” of this sort? That is, how many items can people monitor simultaneously in order to detect an event when it happens? In a series of experiments, we show that people can monitor only two to three items at the same time. This event-monitoring capacity is more limited than the capacity of position tracking (multiple object tracking (MOT)). In the real world, salient cues (e.g., a shout from the crowd) might orient attention to an event. In the absence of such a cue, our results reveal a significant capacity limitation on anyone keeping watch.

## Background

When security personnel watch surveillance videos or monitor the crowds walking on the street, they need to split their attention between multiple things such as pedestrians, vehicles or bikers. In this sort of a task, they are not simply tracking the positions of a set of items, they are looking for classes of events: for example, a suspicious action like a person leaving his bag behind. Little is known about how people perform in this sustained-monitoring task where they have to detect an event in time while monitoring a dynamic scene.

Clearly, how well observers can detect an event in a dynamic scene depends strongly on how many items those observers are able to monitor, unless the event itself summons attention. The ability to divide attention between multiple moving objects has been extensively studied using the MOT task (Pylyshyn & Storm, 1988), where observers are asked to track a set of identical targets moving among identical distractors. Observers are typically asked to track the relevant subset of targets for several seconds. At the end of that time, they might be asked to indicate the position of tracked objects or to declare if a marked item was or was not part of the tracked set. Studies have shown that people are able to accurately track about four items (Cavanagh & Alvarez, 2005; Pylyshyn & Storm, 1988) with variation between different observers (Oksama & Hyönä, 2004) and with the limit changing somewhat with different target variables (Bettencourt & Somers, 2009).

The performance in these experiments, however, mainly reveals a limit of selective attention to otherwise identical items. In the type of event-monitoring task described here, each item in the display could be unique. Therefore, the questions are different. Did a unique item change? Did two different items interact? There is a limited body of research on tracking unique items. Early studies showed that the featural properties of tracked targets are not encoded during MOT (Pylyshyn, 2004; Scholl, Pylyshyn, & Franconeri, 1999). Oksama and Hyönä (2004) asked observers to track visually different line drawing targets (multiple identity tracking, MIT). At the end of each trial, one of the tracked targets was probed and observers were asked to identify the probed target from the presented targets. They found that the targets’ content could be addressed during the position tracking. That is, observers did know, at least to some extent, which target moved where. Similar results were also reported in the tracking of different faces (Ren, Chen, Liu, & Fu, 2009), identities (Horowitz et al., 2007) and color features (Makovski & Jiang, 2009a, 2009b). It has been shown that, during identity tracking, the capacity for localizing the individualized targets was around two (Botterill, Allen, & McGeorge, 2011; Horowitz et al., 2007), which is much smaller than the capacity in position tracking. However, it is still unclear whether the reduced capacity in MIT arises because identity tracking needs to compete for common attentional resources with position tracking (Cohen, Pinto, Howe, & Horowitz, 2011), or whether identity and location tracking are simply governed by two different systems with their own limits (Botterill et al., 2011; Oksama & Hyönä, 2016).

Thus, there are clear limits on the capacity to track objects whether or not they are unique. What about a change in an object or between objects? Even in a static scene, the evidence suggests that multiple event tracking is powerfully limited. Wolfe, Reinecke, and Brawn (2006) asked observers to indicate if any specific dot changed its color from red to green or vice versa. The task was trivial if the color switch was the only visual transient in an otherwise static display. However, if a luminance change also occurred simultaneously with the color change, observers were close to chance performance in deciding if the luminance change was or was not accompanied by a color change. This result does not bode well for the ability to monitor a dynamic scene for the occurrence of an event.

Wolfe et al. (2006) estimated the capacity to monitor a static set of dots to be between 0 and 4, covering the same range as found in MOT and MIT tracking and as found in measures of visual working memory (VWM) capacity (Irwin, 1992; Luck & Vogel, 1997; Wolfe et al., 2006). Indeed, the VWM limitation could be a common limit in all sustained-monitoring tasks. Under many circumstances, detection of change is severely capacity limited (Simons & Rensink, 2005). In the classic version of change blindness, large changes in a scene can be missed if an event, like a blank screen between the original and changed scene, masks the transients produced by the change (Rensink, O’Regan, & Clark, 1997). Under those circumstances, the location of the change is unknown. In the experiments discussed here, observers look for changes in a small, designated subset of the simple stimuli on the screen.

There are only few studies of change detection during MOT. Bahrami (2003) asked observers to track a set of targets among distractors while reporting if there was any color/shape change among them. Observers were able to track all targets and then detect the critical change if the change occurred openly, in the absence of a mud splash to mask the change transient. However, the detection was impaired when the change transition was occluded by mud splashes even if the change occurred in a tracked target. Others have reported that the features of objects are often not encoded during MOT (Pylyshyn, Haladjian, King, & Reilly, 2008; Scholl & Pylyshyn, 1999). It has been suggested that two different systems might be at work during tracking: one would encode the positions of the tracked objects, while the other encodes features and object identity (Horowitz et al., 2007; Oksama & Hyönä, 2016). These systems might still compete for the same attentional resource (Cohen et al., 2011). Thus, if the ability to detect an event among tracked objects shares resources with tracking, performance in event detection might be better when the need for tracking is low.

On the other hand, other phenomena suggest that event monitoring could have a much higher capacity than tracking. Suppose that event detection is similar to a recognition memory task where observers’ task is to distinguish things that have been seen before from novel items. Observers can memorize thousands of specific images and distinguish old from new with good accuracy (Brady, Konkle, & Alvarez, 2011; Brady, Konkle, Alvarez, & Oliva, 2008; Shepard, 1967; Standing, 1973; Standing, Conezio, & Haber, 1970). In a visual search setting, Cunningham and Wolfe asked observers to identify the new object in the visual display. The new item on one trial became an old item for all subsequent trials. Observers could monitor search displays for the new item even when holding a set of hundreds of old items in memory (Cunningham & Wolfe, 2014). Thus, it is possible that the limit on event detection in a sustained-monitoring task might not be limited in the same way that tracking of identical circles is limited.

The goal of the current study is to measure the capacity for detecting events in a sustained-monitoring task. That is, how many items can be monitored at the same time to successfully detect an event when it happens to one of those items? If observers are monitoring a set of otherwise identical objects, waiting for an event to occur, it seems likely that that task will be limited by MOT capacity. However, if items, like individuals in a crowd, are unique, it might be possible in principle to scan through a large number of memorized, unique items, looking for the new event.

To investigate these questions, we used two types of events: in one case, the event was an isolated change occurring to a single item (e.g., the letter T becomes the letter L – e.g., Experiment 1). In the second case, two items interacted with each other, analogous to two people swapping bags (e.g., Experiment 4). To anticipate our results, in all of the variants reported here, observers showed a very limited capacity to monitor for events (capacity *K* = 2–3 items).

## Experiment 1

### Method

#### Participants

Twelve participants (eight women) were recruited from the volunteer pool used by the Brigham and Women’s Hospital’s Visual Attention Laboratory. All had normal or corrected-to-normal vision and passed the Ishihara color screen (or Ishihara color blindness test). Participants gave informed consent approved by the Brigham and Women’s Hospital Institutional Review Board, and were paid US$10/h. Participants ranged in age from 18 to 37 years (*M* = 24.4, *SD* = 6.13).

#### Apparatus and stimuli

**Fig. 1:**
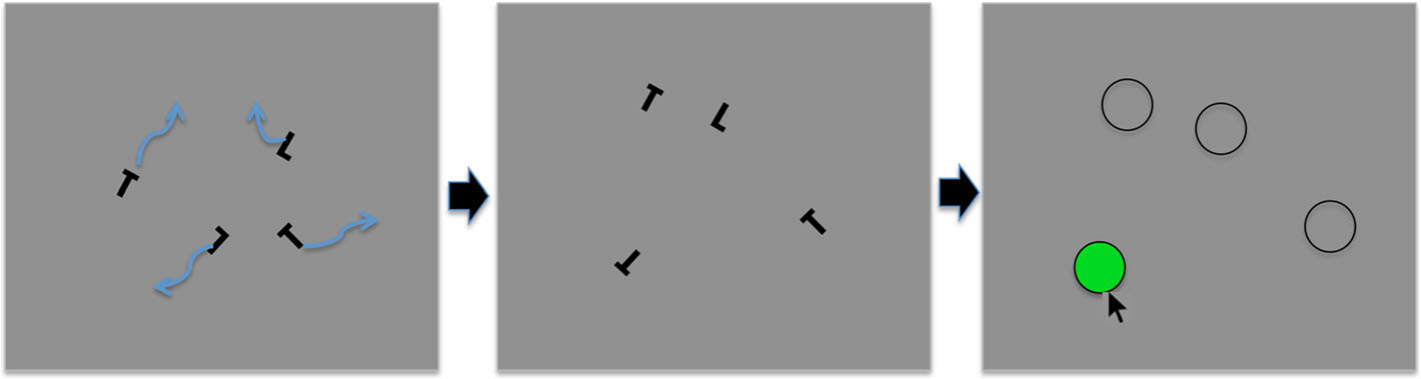
The stimuli and procedure used in Experiment 1. All *N* items were initially stationary for 0.5 × *N* s then started to move. The target would change its identity (T↔L) at a time randomly selected from 0.3 to 6.0 s. Os were asked to respond as soon as they detected the target. Once a response was made, all items would be replaced by empty circles and Os would use the mouse to indicate the prior location of the target. A trial would be terminated if Os did not make a response within 2 s after the change

Stimuli were presented on a 24″ screen on an iMac model A1225 (EMC2211) with resolution = 1920 × 1200. All items would move within an imaginary display of 20° × 20° at a viewing distance of approximately 60 cm. At this viewing distance, 1 cm is nearly equivalent to a visual angle of 1°. The experiments were written in MATLAB 8.3 with Psychtoolbox version 3.0.12 (Brainard, 1997; Kleiner et al., 2007; Pelli, 1997). All items were either black letter Ts or Ls on a gray background (Fig. 1) and the size of each letter was about 0.83° × 0.83°.

#### Procedure

The experiment consisted of four blocks of 50 trials, each with a different set size (*N*) 2, 4, 6 or 8 items. Unlike the conventional MOT task, where selected targets were tracked among distractors, observers were asked to monitor all items until they found the target. At the start of the trial, all items were stationary for 0.5 × *N* s. All items then started to moved with a velocity of 4°/s. When any two items crossed paths, they would overlap. At a time point randomly selected from 0.3 to 6.0 s after the start of motion, the target letter would change its identity from T → L, or vice versa. Observers were told to respond by key press as soon as they found the target. Once observers pressed a key, all items would stop moving and turn into empty circles. Observers would then use the mouse to indicate the location of the target. Accuracy feedback was given after the response was made.

Pilot data made it clear that various guessing strategies could be quite successful. For instance, especially with the smaller set sizes, it is not hard to encode the number of Ts and Ls. If there were more Ts at the end of the trial than at the beginning, the change must have been L → T, so randomly picking a T would produce above chance performance, even though the change was not detected, only inferred. To avoid observers using such a strategy, the trial was terminated and counted as a miss error if observers did not respond within 2 s after the change happened. In addition, for set sizes 6 and 8, there were always at least two Ts and two Ls. To avoid the detection of an abrupt letter change by a transient that could summon attention to otherwise untracked and unattended items, we imposed two different noises: (1) movement noise: all items moved with a small jitter orthogonal to the direction of motion and (2) added transients: every 750 ms all items would change their identity to the letter O for 250 ms, then change back again. These manipulations masked the possible pop-out effect, requiring observers to maintain their focus on as many as items as possible in order to find the target.

#### Data analysis

Our goal was to measure the capacity for event monitoring. How many items can be monitored if a change to one of those items is to be successfully detected within 2 s of the change? Assume that the target event can be detected immediately if it occurs to an item that is being successfully monitored. In this case, the tracking performance (*P*, proportion correct) is given by the number of items actually monitored (*K*, for capacity) divided by the total number of items (set size, *N*). There is no guessing term because of the 2-s response deadline, so:

1 $$ P=\frac{K}{N} $$

Since we know *P* and *N*, we can derive an estimate of *K*.

### Results and discussion

**Fig. 2:**
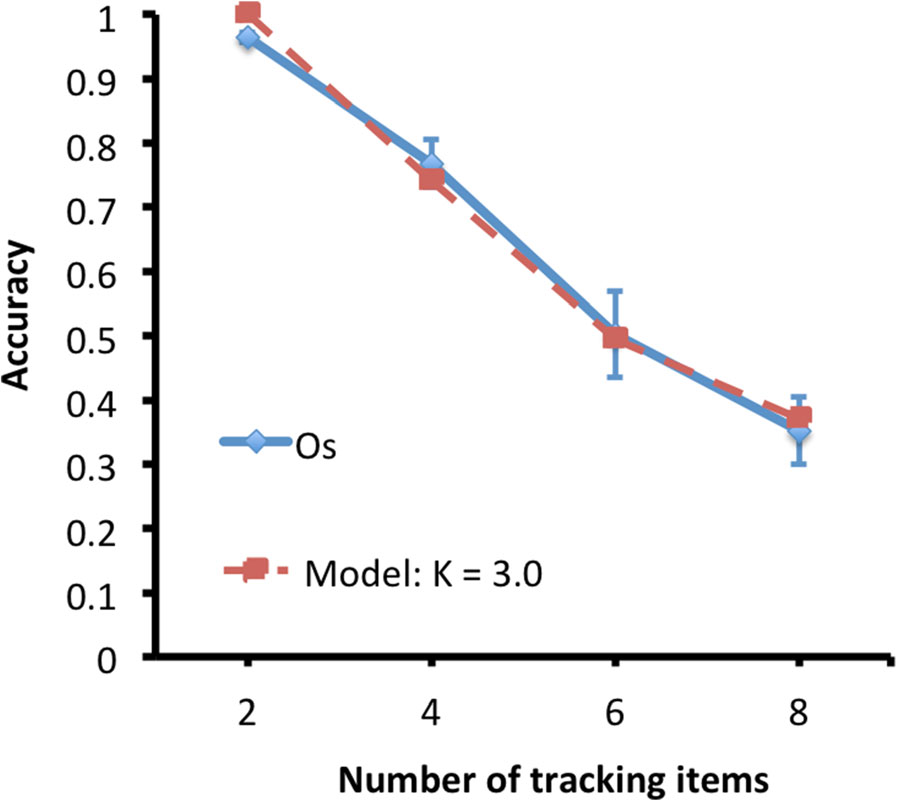
Data from Experiment 1. Tracking accuracy as a function of visual set size. The blue solid line represents observers’ data and the red dashed line represents the model prediction from the estimated capacity (*K* = 3). Error bars are ±1 *SEM*

The event-monitoring capacity was estimated using Eq. 1 for each set size. The resulting estimate of capacity, averaged over set sizes 4, 6 and 8 was 3.4 (set size 2 was excluded since its maximum capacity would be 2 and this would underestimate the overall capacity). However, a further analysis shows that, for the larger set sizes, performance was strongly dependent on the mix of Ts and Ls. The tracking performance was worse when the numbers of Ts and Ls were more evenly distributed. For instance, for set size 8, *P* was 38% when there were four Ts and four Ls. It was 57% when the number of Ts and Ls was unequal. This suggests that observers used a grouping or counting strategy, perhaps choosing to track only the smaller set. To minimize this effect, we only consider the trials where the numbers of Ts and Ls were equal (which was about 43% of the total 2400 trials). The accuracy for these balanced displays is plotted as a function of visual set size in Fig. 2. As expected, the tracking accuracy decreased with set size (a one-way repeated measure analysis of variance (ANOVA), *F*(3,33) = 42.65, *p* < 0.001, ${\eta}_p^2$ = 0.80). The average capacity was about 3.

Even though observers did not need to actually track individual objects in this task, their capacity was, if anything, lower than the approximately four objects that can be tracked in standard MOT paradigms (Pylyshyn & Storm, 1988; Yantis, 1992). In principle, observers could have attended from item to item or group to group checking for change, but whatever their strategy, we found that observers’ capacity for detecting the identity change was relatively small, even at a slow-moving velocity (4°/s).

## Experiment 2

The result of Experiment 1 shows that the capacity for monitoring items for an identity change seems to be lower than the magic number 4 (Cowan, 2001). Perhaps a T becoming an L, or vice versa, is too unnatural. An improbable change might impair performance in change detection (Beck, Angelone, & Levin, 2004). In Experiment 2, we replicated the basic experiment using photorealistic objects that could change their state (e.g., an open bag becomes a closed bag).

### Method

#### Participants

As before, 12 participants (seven women) were recruited from the volunteer pool used by the Brigham and Women’s Hospital Visual Attention Laboratory. The participants ranged in age from 19 to 34 years (*M* = 22.5, *SD* = 4.34).

#### Apparatus and stimuli

**Fig. 3:**
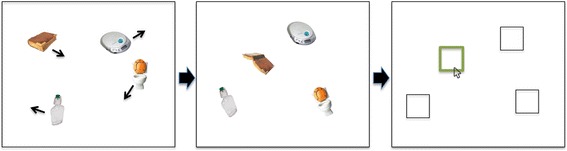
The stimuli and procedure used in Experiment 2. All *N* items were initially stationary for *N* s then started to move. The target would change its state at a time that was randomly selected between the 2nd and 6th second (here, the closed book becomes an open book). Os were asked to respond as soon as they detected the target. Once a response was made, all items would be replaced by empty squares and Os would use the mouse to indicate the prior location of the target. All items would also turn to empty squares after they had moved for 8 s if Os did not make a response

The apparatus in Experiment 2 was identical to that used in Experiment 1. All items in each trial were randomly selected from a set of 31 different objects (Brady et al., 2008). Each of these objects could be portrayed in one of two distinct states (open book versus closed book, as shown in Fig. 3). The size of each item was about 1.89° × 1.89°. The background was white.

#### Procedure

As in Experiment 1, Experiment 2 consisted of four blocks of 50 trials, each with a different set size (*N*) 2, 4, 6 or 8 items. At the start of each trial, all items would remain stationary for *N* s so that observers had sufficient time to encode both the identity and the state of each object. All objects then started to move with a velocity of 4°/s within an imaginary 20° × 20° window. If two objects crossed paths, they would overlap. At a randomly selected time point between the 2nd and 6th second after motion onset, the target object would change its current state to the other state (e.g., open book to closed book). To avoid any pop-out effect caused by a unique transient, all objects sporadically rotated by 30° in one direction for 250 ms and then returned to their original orientation. Observers were asked to respond by key press as soon as they found the target (the object which changed its state). Once the observers responded to the presence of a state change, all objects would stop and were replaced by empty squares. The observers then needed to use the mouse to localize the target. If observers did not make any response, all objects would keep moving for 8 s then turn into empty squares and observers had to guess which one was the target. The feedback was given after the response had been made.

#### Data analysis

As in Experiment 1, performance (*P*) can be estimated from the tracking capacity (*K*) and set size (*N*). However, in this experiment, if observers did not detect the target, they could still guess the target location at the end of the trial. The probability that the target would be one of the *K* tracked objects is $\frac{K}{N}$ and performance is presumed to be 1.0 if the target is tracked. The probability that the target is in the remaining set is $1-\frac{K}{N}$. If this happens, observers would guess the target among the remaining (*N* − *K*) objects. Therefore, performance was given by:

2 $$ P=\frac{K}{N}+\left(1-\frac{K}{N}\right)\left(\frac{1}{N-K}\right) $$

To estimate performance without a guessing component, we can also calculate performance in the manner of Experiment 1, where trials were counted as correct only if observers made a correct response within 2 s after the target changed its state.

### Results and discussion

**Fig. 4:**
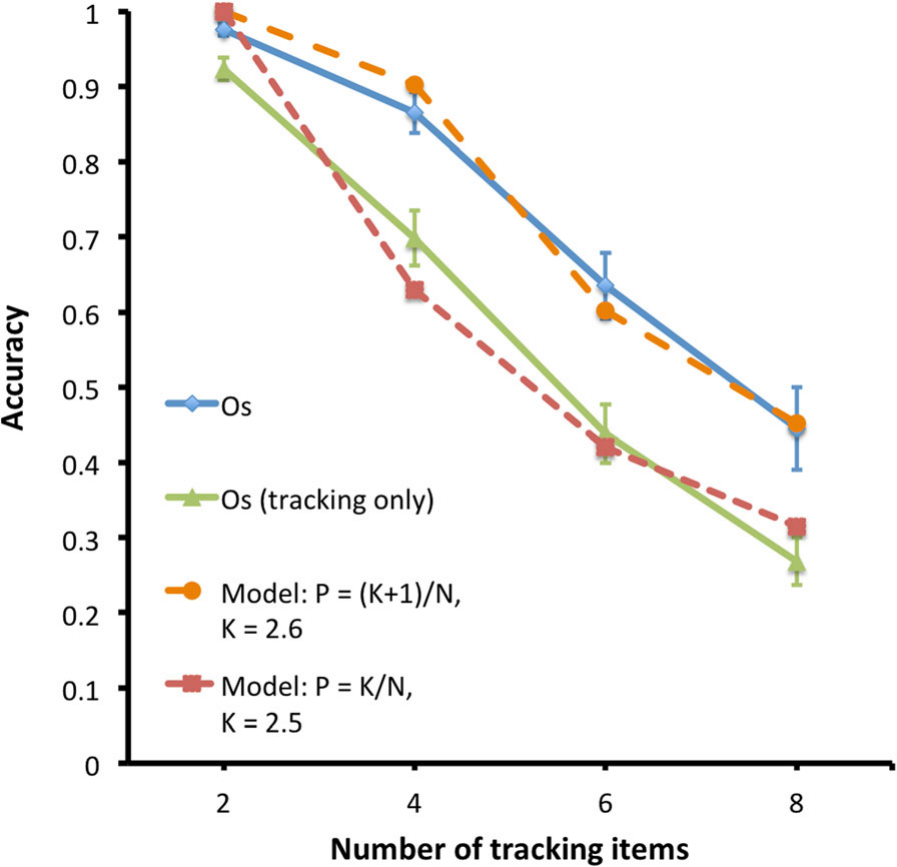
Data from Experiment 2. Tracking accuracy as a function of set size. The blue line represents the raw accuracy in the experiment where Os could track for up to 8 s then guess the location of the target if they had not seen a state change. The green line represents the adjusted performance in which the trials were considered correct only when a correct response was made within 2 s after the target’s state changed. The dashed lines show the estimated performances from the model with guessing (*orange*) and without guessing (*red*). Error bars are ±1 *SEM*

As in Experiment 1 and as shown in Fig. 4, tracking accuracy decreased as a function of set size (*F*(3,33) = 61.48, *p* < 0.001, ${\eta}_p^2$ = 0.85). By using Eq. 2 and the raw accuracy, the estimated capacity (averaged from set size 4 to 8) was 2.6 items. Notice that this estimate of capacity might contain two factors; an ability to detect a change when it happened and an ability to remember the initial state of some number of objects and to notice that one object was now in a different state, even if the moment of change was not detected. Computing the accuracy in the same way as in Experiment 1 by requiring a response within 2 s of the change should minimize the memory-checking component and emphasize the on-line monitoring component.

Figure 4 shows that performance decreased with set size as expected (*F*(3,33) = 110.15, *p* < 0.001, ${\eta}_p^2$ = 0.91). Raw performance with the 2-s deadline is necessarily worse than performance that permits some successful guessing. Interestingly, the best-fitting model has a value of *K* of 2.5, not notably different from the 2.6 estimated with the guessing correction. This suggests that the memory component was of little use to participants in Experiment 2.

## Experiments 3 and 4

The previous two experiments focus on the changes of a single entity. Our results show that observers were able to track more than one item when looking for an abrupt change, but the capacity was low. Experiments 3 and 4 consider the situation in which we are monitoring for an interaction between entities (e.g., did this person hand his backpack over to that other person who just passed by?). How many agents can people monitor when looking for this kind of interactive event? The following two experiments were designed to answer this question.

### Method

#### Participants

Under the same conditions as in Experiments 1 and 2, 12 observers (seven women) and 13 observers (eight women) were recruited from the Visual Attention Laboratory’s volunteer pool for Experiment 3 and Experiment 4, respectively. Participants ranged in age from 18 to 52 years (*M* = 26.75, *SD* = 9.21 in Experiment 3 and *M* = 28.92, *SD* = 11.25 in Experiment 4).

#### Apparatus and stimuli

**Fig. 5:**
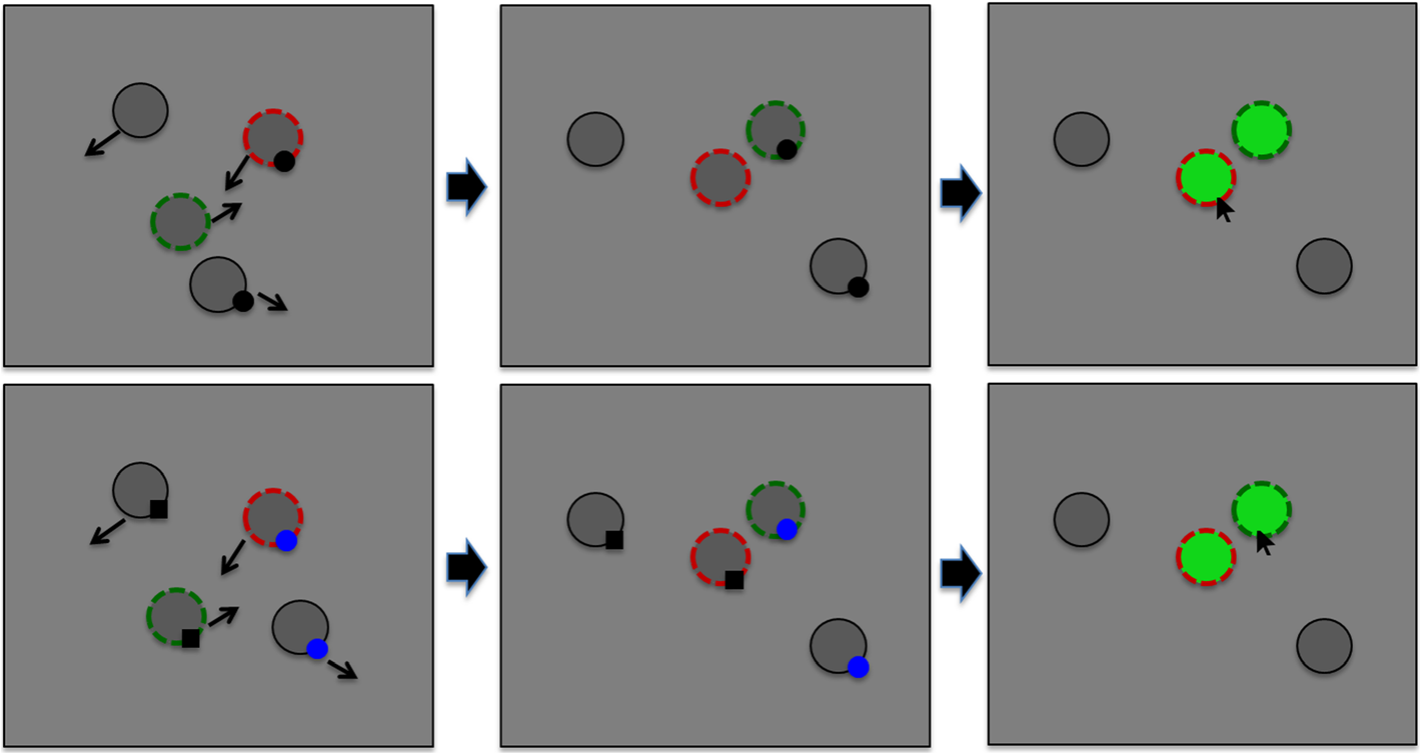
The stimuli and procedure used in Experiment 3 (top) and Experiment 4 (bottom).All items were stationary at the start of the trial, then started to move. At a certain point, one item (here shown with a red outline that was not present in the experiment) would pass/exchange its bag to another item (green outline). Observers had to make a detection response within 2 s after the event occurred. The observer then used the mouse to indicate the locations of both passer and receiver

The apparatus used in Experiments 3 and 4 was the same as in the previous experiments. The items being tracked were identical, dark-gray circles with black outlines placed on a light gray background. In Experiment 3, half of the circles had a small, solid, black circle attached to them as a simulation of an object held by an agent (see Fig. 5). In Experiment 4, half of the circles had an attached blue circle and the other half had an attached black square to simulate two groups of agents holding different objects that could be exchanged.

The positions of the same type of bags were identical in a given trial. Thus, in Fig. 5, all bags were attached to the lower right side of a bigger circle. This position was varied trial by trial. The size of each item was about 1.2° × 1.2° and the size of each bag was about 0.5° × 0.5° at an approximately 60-cm viewing distance.

#### Procedure

In Experiment 3, observers were asked to detect an event where an agent would “pass” their bag to another, empty-handed agent. In Experiment 4, observers were asked to detect the event where the agent holding a blue bag would “exchange” their bag with another agent holding a black bag. As in the previous two experiments, both experiments consisted of four blocks of 50 trials, each with a different tracking set size (*N*) 2, 4, 6 or 8. Note that the number of items shown in each display was twice as big as the effective tracking set sizes because observers only needed to track half of the items in order to detect the event. For instance, if the tracking set size was 4 in Experiment 3, there would be four items holding a black bag and another four items without a bag. To detect the event, observers only need to monitor those four items with/without a bag rather than track all the items on the screen.

As in previous experiments, at the start of a trial, the *N* items were stationary for *N* s. All items then started to move in straight lines, except when they bounced off the side of an imaginary window (20° × 20°). At a certain point in time, 0.15–4 s after the start of motion, one circle would pass its bag to another circle which did not have a bag (Experiment 3), or exchange its bag with another circle with a different type of bag (Experiment 4). In addition, to make a smooth pass or exchange, the event only occurred when the distance of the two interacting items was smaller than the circles’ radius. Observers were asked to make a response by key press as soon as they detected the pass/exchange event. If observers did not respond within 2 s after the event occurred, the trial would be terminated and counted as a miss. Once the detection response was made, all items became identical circles (i.e. all bags were removed from the display) and observers would use the mouse to indicate which two items were the passer and receiver in Experiment 3, or which two items exchanged their bags in Experiment 4.

#### Data analysis

In each trial, observers needed to indicate both items involved in the event. Thus, accuracy was the numbers of correct items selected divided by two. This meant that it was possible for an observer to be half right if only one of the two items was correctly marked. Accuracy was considered to be zero if the trial was a miss (i.e. when observers did not respond within 2 s after the event). As in Experiment 1, the 2-s deadline minimized guessing, so the overall tracking performance *P* is given by Eq. 1 (*P* = *K*/*N*).

### Results and discussion

As expected, the tracking accuracy in both experiments decreased as a function of tracking set size (*F*(3,33) = 219.4, *p* < 0.001, ${\eta}_p^2$ = 0.95 for Experiment 3; *F*(3,33) = 292.94, *p* < 0.001, ${\eta}_p^2$ = 0.96 for Experiment 4). To compare performance between the two experiments, we conducted a two-way, mixed design ANOVA with experiment type (“pass” or “exchange”) as a between-subject factor and set size as a within-subject factor. The result shows that there was no difference in performance between Experiment 3 and Experiment 4 (*F*(1,23) = 0.94, *p* = 0.34). The estimated capacities were about 2.6 in Experiment 3 and 2.8 in Experiment 4 (averaged capacities from set sizes 4, 6 and 8, as before).

Similar to the results when detecting an isolated event in the previous two experiments, the capacity for detecting the interactive event was also quite limited. It is possible that the low capacity found in Experiment 3 and Experiment 4 was due to the confusion between the *K* tracked items and (*N* – *K*) untracked items in the same set. Since all items were identical, observers may not be able to retrieve a selected item if they lost track of it. To examine this possibility, we used items with unique identities in Experiment 5.

## Experiment 5

The results of Experiments 3 and 4 show that the capacity to detect an interaction between two items is low. The use of identical items may contribute to the low capacity since it is likely that observers sometimes confused tracked items with untracked items within the same set of objects. In the real world, (e.g., monitoring activity in a crowd), the items of interest would not be identical. Accordingly, in Experiment 5, we replicated the interaction experiment, using items that each had a unique identity. Horowitz et al. (2007) had shown that providing unique identity can improve MOT (albeit not dramatically). Therefore, if the low capacity for detecting an interaction event was due to a swapping error between tracked items and untracked items in the same set, performance should be improved when all items are unique.

### Method

#### Participants

Twelve participants (eight women) were recruited from the Visual Attention Laboratory’s volunteer pool, as described above. Participants ranged in age from 18 to 33 years (*M* = 24.92, *SD* = 5).

#### Apparatus and stimuli

**Fig. 6:**
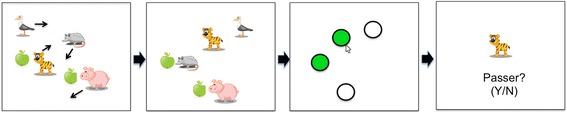
Stimuli and procedure used in Experiment 5. At the start of the trial, all items were stationary for *N* s and then started to move. If and when observers detected one animal passing its apple to another animal, they would respond immediately and then use the mouse to indicate which two items were involved in this event. After that, observers were shown an animal and asked whether it was the animal that had passed its apple to another

The apparatus and stimuli for Experiment 5 were similar to those used in Experiment 3 except that each identical item was replaced by a unique cartoon animal. In each trial, all items were randomly selected from a set of 25 different cartoon animals. Items subtended about 1.4° × 1.4° at an approximately 60-cm viewing distance. In each trial, half of the animals carried a green apple (0.5° × 0.5°) on their left side (Fig. 6). The background was white.

#### Procedure

The procedure in Experiment 5 was identical to Experiment 3 except that, after indicating the locations of both the passer and the receiver, a follow-up question was asked about the identity of the animal passing the apple. Observers were shown an animal and asked if it had passed the apple. Observers were shown the correct animal on 50% of trials. Feedback for the trial was presented once they made this response. No identity task was given if the trial was a miss.

### Results and discussion

**Fig. 7:**
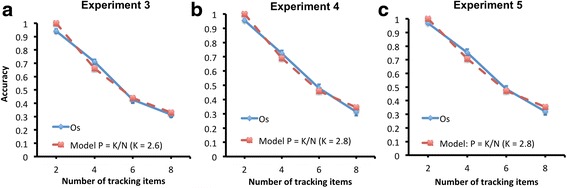
Data from Experiment 3 to Experiment 5. Tracking accuracy as a function of set size. The blue lines represent observers’ tracking accuracy. The red lines represent the prediction of the model. Error bars are ±1 *SEM*

Figure 7 shows that Experiments 3, 4 and 5 produce comparable results. Similar to the results in the previous experiments, tracking accuracy declined as a function of tracking set size in Experiment 5 (*F*(3,33) = 312.78, *p* < 0.001, ${\eta}_p^2$ = 0.96). The accuracy of responses to the final, passer identification question was 84% (*SD* = 14%). Interestingly, as shown in Fig. 7, adding unique identity to each item did not affect the tracking performance. A mixed-design ANOVA with experiments as a between-subject factor and set sizes as a within-subject factor did not reveal a significant effect of experiment, *F*(2, 34) = 1.22, *p* = 0.31).

Unlike other experiments, where it was found that the unique identity of objects could improve MOT (Horowitz et al., 2007; Makovski & Jiang, 2009a), we did not find the same advantage in our event-monitoring tasks. One may argue that the results were obtained by comparing performances across different groups of observers. It is possible that the advantage of unique identity was hidden by the variation between different groups of observers. Moreover, our observers had to detect the interaction event while continuing to encode/update objects’ identities and positions. It is possible that the identification and event-detection tasks may have interfered with each other. We tested this possibility in Experiment 6 by having a single group of observers conduct identity tracking and event tracking in separate blocks.

## Experiment 6

In this Experiment, each observer participated in three blocks of trials: the identity tracking task, the event-monitoring task, and both tasks together (the combined condition replicates Experiment 5). If the results of Experiment 5 were influenced by between-subject variability or the Dual-task load, then event-monitoring performance should improve when there is no need to encode the identity.

### Method

#### Participants

Twelve participants (six women) were recruited from the Visual Attention Laboratory’s volunteer pool, as described above. Participants ranged in age from 18 to 47 years (*M* = 27.42, *SD* = 10.65).

#### Apparatus and stimuli

The apparatus and stimuli used in Experiment 6 were identical to those used in Experiment 5.

#### Procedure

**Fig. 8:**
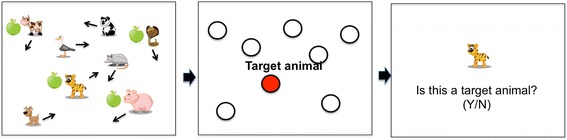
The stimuli and procedure for the Identity-only condition of Experiment 6. At the start of each trial, all items were stationary for 8 s and then started to move. Observers were asked to track the identities of the animals that held the apple. At a time selected from the distribution of times for the passing event in Experiment 5, all animals would stop moving and then become identical circles. One of the circles that had originally been an animal holding the apple would be probed. Observers were tested on the identity of the animal that had been at the probed location

The aim of Experiment 6 was to test whether the need to encode identity for later report interfered with the event monitoring in Experiment 5. To dissociate the memory component, we tested one group of observers with displays of eight total items on the screen – an effective tracking set size of *N* = 4. There were 50 trials in each of three separate blocks: (1) Identity-only (shown in Fig. 8), (2) Event-only and (3) Dual-task. The effective tracking set size was 4 for all three blocks.

In the Identity-only condition, the procedure is similar to Experiment 5, but no pass event occurred. Observers were asked to track the animals holding the apple. The movement duration was sampled from the distribution of event times in Experiment 5. That is, one of the times when an event occurred in Experiment 5 was chosen as the duration of movement in the Identity-only condition of Experiment 6. Once the movement stopped, the unique animals became empty circles. One circle was probed and the observer was given a two-alternative forced choice (2AFC) Y/N option about the identity of the animal that had been at that location. Observers responded with a key press after which feedback was presented.

In the Event-only condition, the procedure was identical to the one in Experiment 5, but with no identity test at the end. Observers only needed to detect the passing of an apple. Then, as in Experiment 3, they indicated which two items were the passer and receiver. In the Dual-task condition, the same group of observers was tested in a direct replication of Experiment 5. The order of blocks was counterbalanced.

### Results and discussion

**Fig. 9:**
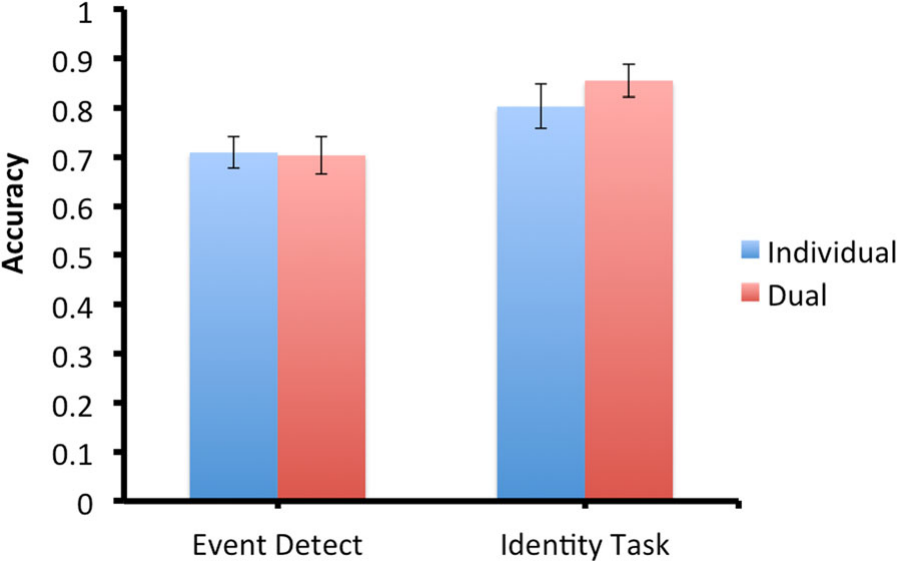
The data for Experiment 6. The left-hand bars represent the accuracy of event detection in the individual task (*blue*, condition 1) and the Dual-task (*red*, condition 3). The right-hand bars represent the accuracy of identification in the individual task (*blue*, condition 2) and the Dual-task conditions (*red*, condition 3). Error bars are ±1 *SEM*

Figure 9 shows performance in the event-monitoring and identification tasks. There was no difference in the event-monitoring performance between the individual task and Dual-task conditions (pairwise *t* test: *t*(11) = 0.23, *p* = 0.83). That is, the need to encode and report the identity of the passing animal does not appear to affect event detection. Similarly, there was no difference found in identity task performance in the individual versus Dual-task conditions (*t*(11) = 1.47, *p* = 0.17).

The results of Experiment 6 show that the low capacity for event-monitoring in the previous experiment was not due to the different groups of observers or to Dual-task effects.

## General discussion and a control experiment

One might hope that it would be possible to look at a crowd of individuals, milling about in the town square, and to notice if one of them did something odd or if two of them exchanged a suspicious package. The results of the experiments presented here suggest that this ability has a very limited capacity. It is not possible to monitor large numbers of items at the same time in a sustained event-monitoring task. Observers might select a limited set of items to track or they might scan through the whole set, only effectively attending to a few items at any given time and hoping to catch the change if it happens to an attended item.

**Fig. 10:**
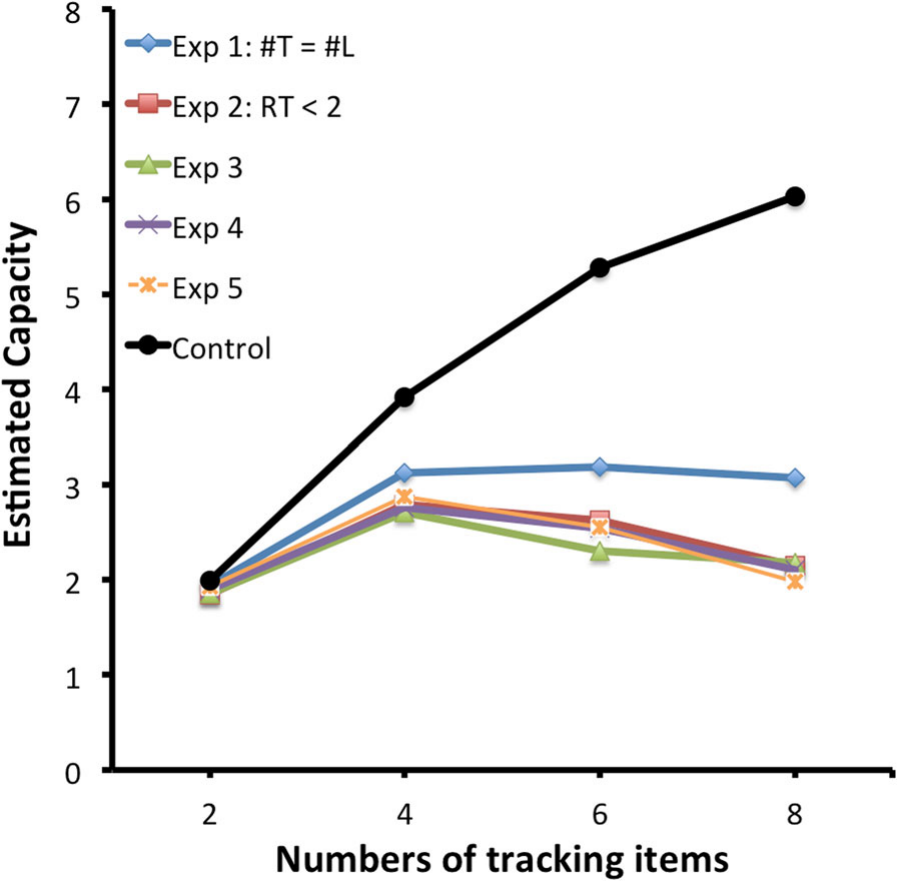
Estimated capacity as a function of tracking set size in the event-detection and control tasks

Our results show that this event-monitoring capacity is very limited (two to three items). This is lower than typical estimates of the capacity for Multiple Object Tracking (MOT). Note that even though the speed of movement in the present study was quite modest (approximately 4°/s), it might still be faster than the regular movements of people in a real scene. This, of course, would depend on factors such as the viewing distance. Alvarez and Franconeri (2007) have shown that the tracking capacity in MOT can vary from one to eight items depended on motion speed. Thus, it is possible that we simply chose conditions that produced low MOT performance and that poor performance on the event-monitoring task simply reflects poor MOT performance, even in conditions where the tracked set was persistently marked (e.g., by apples in Experiments 5 and 6). To assess this possibility, we conducted a simple MOT control experiment in which observers viewed the same stimuli as in Experiment 5 but without an apple-passing event. Observers were asked to track the animals holding an apple. The tracking duration was sampled from the passing times used in Experiment 5. Once the movement stopped, all animals were replaced by identical circles and observers were asked to use the mouse to indicate the locations of all target animals. The results of this control experiment give an estimate of the maximum number of items that could be tracked in our experiments when each item is persistently distinct from distractors during the tracking period. Figure 10 shows the estimated capacities in the control task compared to the event-monitoring capacities, measured in Experiments 1 to 5. Clearly, observers were able to track more than five items in our setup. However, when event monitoring was required, the capacity was limited to two to three items.

The purpose of the present study is to examine how many items people can attend to at the same time in order to reliably detect an event when it occurs. To what extent does this ability depend on the nature of the change? In the first two experiments, observers were asked to detect a change in the state of an individual item. In the other experiments, observers were asked to detect an interaction between two items. The interaction events might be monitored by a variety of strategies not available for the single-item events. For instance, observers might try to attend to impending close contact between items since those were the moments at which an exchange could occur.

Comparing reaction times (RTs) between different tasks might be a window into differences in strategy. In comparing RTs, we have tried to exclude strategic factors that might artificially boost apparent event-monitoring capacity. In Experiment 1, we only considered the trials with equal numbers of Ts and Ls, so as to minimize the effect of attending to the smaller group. In Experiment 2, we only included trials with responses occurring within 2 s after the change occurred. If observers shifted their attention from item to item, RTs should increase with the tracking set size. On the other hand, if observers monitored a set of *K* items at the same time, then the RTs might be similar across all set sizes smaller than *K*.

**Fig. 11:**
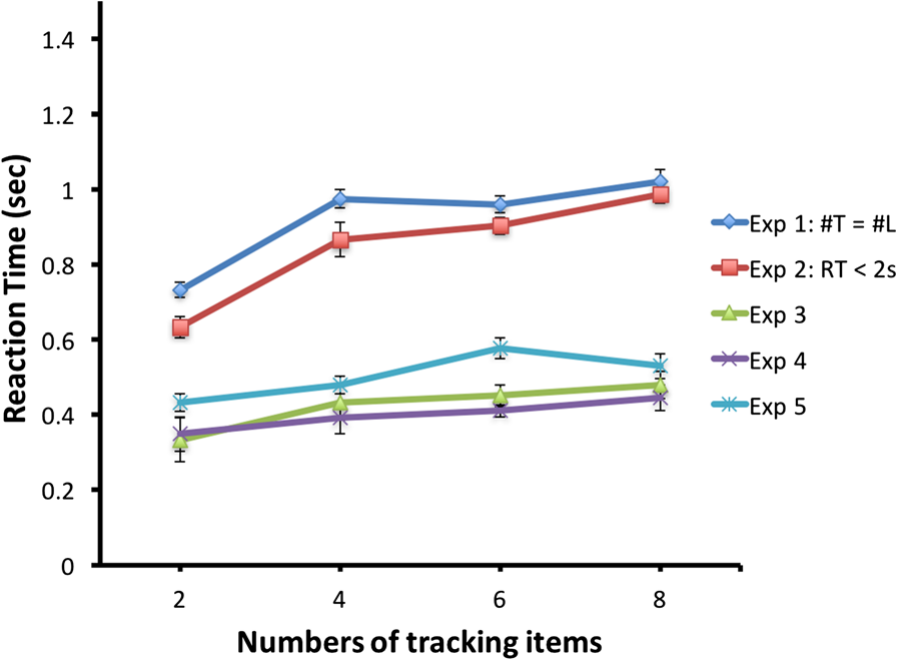
Reaction time as a function of tracking set size in Experiment 1 to Experiment 5. Error bars are ±1 *SEM*

Figure 11 shows RT as a function of tracking set size across different tasks. When looking for an isolated event, RTs increased with tracking set size (59 ms/item), suggesting that observers shifted their attention from one item to another. When observers monitored for an interaction between two items, the tracking set size only had a small effect on RTs (16 ms/item), suggesting that observers adopted very different viewing strategies due to the different nature of change (a two-way ANOVA showed a significant main effect of experiment type, *F*(4,220) = 123.54, *p* < 0.05, ${\eta}_p^2$ = 0.69). It is interesting that detecting the interactions produced faster responses than detecting the individual event. Thornton, Bülthoff, Horowitz, Rynning, and Lee (2014) found a comparable result in which people could control more items in a collision-avoidance task than they could monitor in a tracking task. A similar effect could be occurring here. However, the present set of experiments does not distinguish candidate differences between the interaction and state-change tasks. It could be that near collisions allow anticipation of an interaction event or differences in RTs could be due to differences in perceptual difficulty (i.e. the change from closed to open book might just take longer to process than the movement of an apple). There might be other causes. Regardless, in all cases, event monitoring appears to have a similar range of capacity (two to three items, Fig. 10) even though the RTs can be quite different.

To perform a sustained-monitoring task of the sort described in this paper, people have to register the initial and current status of some subset of items. That is, observers cannot know if an entity has dropped a bag unless they knew that the entity had a bag in the first place. Therefore, the capacity to monitor events could be limited by the number of items people can remember in the first place. It has been shown that the visual working memory (VWM) capacity is very similar to MOT capacity (Luck & Vogel, 1997). Thus, one could propose that VWM was the limiting factor in these experiments. If that were the case, higher memory capacity might lead to a better event tracking performance (though we did not test this possibility). It is more likely that the relationship between VWM and event-monitoring capacity will be a relatively complicated interaction with motion-tracking capacity. Tracking the identities of a set of moving items is more limited than simply tracking a set of undifferentiated items. The apparent difficulty that observers have with location-identity binding during tracking (Horowitz et al., 2007) may explain the relatively low capacities we see in these experiments and might serve to decouple those capacities from standard VWM capacities.

Interestingly, when the observers were asked to report the passer’s identity (Experiments 5 and 6), the RTs became somewhat longer, but tracking performance was not affected. Moreover, the increase in RTs seems too short to support a strategy of processing the identity of the passer information only after the event was detected (+80 ms in Experiment 5 and +91 ms in Experiment 6). This suggests that observers were aware of the identity of the target items prior to the event. A memory load interferes with standard MOT (Fougnie & Marois, 2006), but adding the requirement to remember target identity does not seem to affect event monitoring in our experiments.

In the current experiment, we did not consider the effect of object spacing on event detection. Franconeri, Jonathan, and Scimeca (2010) found that the main constraint in MOT is object spacing and they concluded that the tracking capacity can be unlocked if there was no object-spacing constraint. In our sustained-monitoring task for an interactive event, the numbers of no-need-to-track items (e.g., the numbers of animals without the apple) increased with tracking set size. It is possible that the modest decline in estimated capacity at higher set sizes is due to increased crowding or other effects of spacing (see Fig. 10).

## Conclusions

In summary, the sustained-monitoring task is a real-world challenge. Returning to the opening example, we would like to believe that the guard keeping watch could detect a critical event in time to save us from some significant cost. The results of this study are sobering, suggesting that people can monitor only two to three items at a time. This capacity seems to be similar, but not the same, as the limits on position tracking in MOT or the contents of VWM.
